# Antioxidant Sol-Gel Improves Cutaneous Wound Healing in Streptozotocin-Induced Diabetic Rats

**DOI:** 10.1155/2012/504693

**Published:** 2012-08-07

**Authors:** Yen-Hsien Lee, Jung-Jhih Chang, Chiang-Ting Chien, Ming-Chien Yang, Hsiung-Fei Chien

**Affiliations:** ^1^Graduate Institute of Applied Science and Technology, National Taiwan University of Science and Technology, Taipei 106, Taiwan; ^2^Department of Materials Science and Engineering, National Taiwan University of Science and Technology, Taipei 106, Taiwan; ^3^Department of Medical Research, National Taiwan University Hospital and National Taiwan University College of Medicine, Taipei 100, Taiwan; ^4^Department of Surgery, National Taiwan University Hospital and National Taiwan University College of Medicine, Taipei 100, Taiwan

## Abstract

We examined the effects of vitamin C in Pluronic F127 on diabetic wound healing. Full-thickness excision skin wounds were made in normal and diabetic Wistar rats to evaluate the effect of saline, saline plus vitamin C (antioxidant sol), Pluronic F127, or Pluronic F127 plus vitamin C (antioxidant sol-gel). The rate of wound contraction, the levels of epidermal and dermal maturation, collagen synthesis, and apoptosis production in the wound tissue were determined. *In vitro* data showed that after 6 hours of air exposure, the order of the scavenging abilities for HOCl, H_2_O_2_, and O_2_^−^ was antioxidant sol-gel > antioxidant saline > Pluronic F127 = saline. After 7 and 14 days of wound injury, the antioxidant sol-gel improved wound healing significantly by accelerated epidermal and dermal maturation, an increase in collagen content, and a decrease in apoptosis formation. However, the wounds of all treatments healed mostly at 3 weeks. Vitamin C in Pluronic F127 hastened cutaneous wound healing by its antioxidant and antiapoptotic mechanisms through a good drug delivery system. This study showed that Pluronic F127 plus vitamin C could potentially be employed as a novel wound-healing enhancer.

## 1. Introduction

Wound healing represents a well-orchestrated reparative response that occurs after all surgical procedures or traumatic injury. Wound healing is a complex multifactorial process, involving inflammation, migration of different cell types, fibroplasia, collagen deposition, and wound contraction. During the inflammation phase, inflammatory cells significantly increased in the wound site [1] and produced burst amounts of reactive oxygen species (ROS) formation in the wound tissue [2] affecting wound healing.

Diabetes mellitus is one of the major contributors to chronic wound-healing problems, because minor skin wounds can lead to chronic, nonhealing ulcers and ultimately result in infection, gangrene, or even amputation. In critical ill diabetic patients, the antioxidant vitamin C in plasma was reported lower than nondiabetic critical ill patients [3]. In streptozotocin-induced and gene-induced diabetic mice, increased oxidative stress in the wounds has been noted [2]. Besides, increased oxidative stress promoted apoptosis formation in the damaged tissue and the increased apoptosis signaling also delayed the wound-healing process [4]. In addition, the diabetic rat skin was underhydroxylated in nascent collagen alpha chains (types I and III) [5]. Compromised collagen production associated with vitamin C deficiency results in impaired wound healing [6].

Vitamin C is an important water-soluble antioxidant, which may successfully scavenge ROS, protect against lipid damage, protein oxidation, and DNA oxidation [7]. Vitamin C can overcome the reduced proliferative capacity of elderly dermal fibroblast as well as increasing collagen synthesis in elderly cells [8]. Vitamin C promotes the hydroxylation, which is required to stabilize the triple helical conformation of collagen [9]. Silvetti [10] has presented a safe and effective method of improving repair and controlling infection of wounds by daily topical application of a balanced solution of salts, amino acids, a high-molecular weight, D-glucose polysaccharide, and vitamin C. Vitamin C is water soluble but it is the most unstable of all water-soluble vitamins. Vitamin C reacts with the metallic ions of iron and copper and is easily destroyed by oxygen, alkalies, and high temperature [11].

Pluronic F127 is one member of a family of triblock copolymers of poly(ethylene oxide)-poly(propylene oxide)-poly(ethylene oxide), generically called poloxamers. At low temperatures, poly(propylene oxide) blocks have only weak hydrophobic properties. With increasing temperature, poly(ethylene oxide) blocks are dehrydrated and promote the aggregation to micelles and become gel form [12]. The incorporation of drugs into Pluronic micelles results in enhanced metabolic stability because of the outer hydrophilic poly(ethylene oxide) chains that protect drugs from external conditions. Pluronic F127 in a gel form has been used previously as a wound dressing [13] and as a drug delivery vehicle [14, 15]. In this study, we aimed to develop an antioxidant sol-gel preparation by incorporating vitamin C into Pluronic F127 and to apply the antioxidant sol-gel on cutaneous wounds in normal or diabetic rats. The parameters of wound closure rate, epidermal and dermal maturation, collagen synthesis, and apoptosis formation in wounds were evaluated. We hypothesized that the antioxidant ability of vitamin C in Pluronic F127 sol-gel was better than in saline solution, and antioxidant treatment will improve cutaneous wound healing in diabetic rats.

## 2. Materials and Methods

### 2.1. Animals

Female Wistar rats (*n* = 6 in most comparing group, *n* = 5 in ROS measurement), weighing 180 ± 20 g, were used to experiment. All rats were housed at a constant temperature and humidity in a room with an artificial 12-h light/dark cycle and allowed free access to food and water. All the surgical and experimental procedures were approved by Institutional Animal Care and Use Committee of National Taiwan University College of Medicine and College of Public Health and were in accordance with the guidelines of the National Science Council of Republic of China (NSC 1997).

### 2.2. Preparation of Antioxidant Sol-Gel

Pluronic F127 and vitamin C were purchased from Sigma-Aldrich Chemical Co. (USA). The Pluronic F127 (13% w/w) was dissolved in saline at 4°C by stirring into homogeneous sol-gel. Vitamin C powder was dissolved in the Pluronic F127 sol-gel (1 mg vitamin C/mL Pluronic F127 solution) as antioxidant sol-gel. The vitamin C powder was dissolved in saline (1 mg/mL) as vitamin C solution. We compared the *in vitro* antioxidant activities of 4 groups, that is, the saline, saline plus vitamin C (antioxidant saline), Pluronic F127, and Pluronic F127 plus vitamin C (antioxidant sol-gel) in this study. For this study, 200 *μ*L samples exposed at 1 cm^2^ area were determined their antioxidant ability at 0 and 6 hours after exposure to the air.

### 2.3. Measurement of ROS and Antioxidant Abilities [16]

To measure the production of ROS in the samples, chemiluminescence (CL) method was adopted using lucigenin (0.25 mM) as an amplifier for measuring superoxide (O_2_^−^) and luminol (0.25 mM) as an amplifier for measuring hydrogen peroxide (H_2_O_2_) and hypochlorous acid (HOCl). In brief, 0.2 mL of the samples (homogenized skin biopsies, vitamin C solution, Pluronic F127 sol, or antioxidant sol-gel) was placed in the plate for oxidative stress assay using a CL analyzer (Top Count System; Packard, Meriden, CT, USA). For H_2_O_2_ measurement, the sample and 0.5 mL of luminol were added on the dish, and the photon emission from the sample was count at 60-sec intervals at room temperature under atmospheric conditions. For measuring antioxidant abilities, after 120-sec incubation, 0.1 mL of 1 mM H_2_O_2_ was added. For hypochlorous acid (HOCl) measurement, the sample and 0.5 mL of luminol were added on the dish, and the photon emission from the sample was counted as previously. For measuring antioxidant abilities, after 60-sec incubation, 0.1 mL of 1 mM HOCl was added. For superoxide (O_2_^−^) measurement, the sample and 0.5 mL of 0.25 mM lucigenin were added on the dish, and the photon emission from the sample was counted as before. For measuring antioxidant abilities, after 60 sec incubation, 0.1 mL of 0.15% xanthine and 0.1 mL of 0.6% xanthine oxidase were added. For each sample, the assay was performed in triplicate, and the reactive oxidant level was expressed as CL counts. 

### 2.4. Induction of Diabetic Rats and Wounding

Diabetes was induced by a single 65 mg/kg intraperitoneal injection of streptozotocin (STZ; Sigma, Inc., St. Louis, MO, USA), a toxin specific for insulin-producing cells, in normal saline. Blood glucose levels were measured using an acute glucometer. The diabetic state was confirmed 3 weeks after STZ injections by blood glucose levels above 300 mg/dL. Under brief anesthesia with intraperitoneal Nembutal (65 mg/kg), the dorsal skin of the animals was shaved and cleaned with povidone-iodine solution, and a full-thickness skin wound (approximately 1 × 1 cm^2^) was created after marking the area with a wooden ink stamp before cutting the outlined skin. We applied 0.2 mL of antioxidant sol-gel, antioxidant saline, Pluronic F127, or saline on wounds twice per day for 21 days. Wound size was recorded with photographs, after anesthesia each time at 0, 7, 14, and 21 days after wounding. The wound size was then calculated with a free program called Image J. Animals were euthanized at each time point and the wound samples and adjacent normal skin were harvested and fixed in 10% paraformaldehyde for histological or snap-frozen in liquid nitrogen and stored at −80°C for further analysis. For detecting skin ROS, the intact skin (0.5 g) of diabetic rats as well as normal rats was biopsied, snap-frozen in liquid nitrogen, and homogenized by mortar and pestle, followed by adding 1 mL normal saline.

### 2.5. Wound-Healing Rate

The percentage of wound closure was calculated as follows by using the initial and final area drawn on glass slides during the experiments:

(1) $$\begin{array}{c} \%\;\text{of}\;\text{wound}\;\text{contraction}=\frac{A_{0}-A_{t}}{A_{0}}\times 100\%, \end{array}$$

where *A*_0_ is original wound area and *A*_*t*_ is the area of wound at days 7, 14, and 21, accordingly.

### 2.6. Hydroxyproline Analysis [17]

Wound tissues stored at −80°C were dried to a constant weight and hydrolyzed in 6 M HCl for 16 h at 120°C. Samples were dried on a hot plate and then washed three times with distilled water. The acid-free samples were reconstituted in 2.0 mL of acetate-citrate buffer (1.2% sodium acetate trihydrate, 5% citric acid, 12% sodium acetate, and 3.4% sodium hydroxide, pH 4–9). Five hundred microliters of 0.05 M chloramine-T was added to 1 mL of each sample, after which the samples were incubated for 15 min at room temperature, followed by the addition of 0.5 mL 15% perchloric acid and 15% 4-dimethyl aminobenzaldehyde in 1-propanol. After incubation at 60°C for 15 min, each sample was transferred to a microliter plate and the absorbance read at 550 nm. Hydroxyproline concentrations were calculated from the linear standard curve and presented as *μ*g/g dry tissue weight.

### 2.7. Histological Analysis

#### 2.7.1. Epidermal and Dermal Maturation Assessment

Wound bed biopsies were collected at days 7, 14, and 21 after wounding. Tissue samples were fixed in 10% buffered formalin, processed, and embedded in paraffin. Sections were stained with hematoxylin and eosin (H & E). Microscopic assessment of these slides was coded by a technician, and read-blinded to the sample identification. The sections were scored on a scale of 0–4 for epidermal healing (0 = no migration, 1 = partial migration, 2 = complete migration with partial keratinization, 3 = complete keratinization, and 4 = hypertrophic epidermis) and dermal healing (0 = no healing, 1 = inflammatory infiltrate, 2 = granulation tissue present-fibroplasias and angiogenesis, 3 = collagen deposition replacing granulation tissue > 50%, and 4 = hypertrophic fibrotic response) [18].

To investigate this further, differentiation of the neo-epidermis was studied by immunohistology using loricrin as late differentiation marker. Structural proteins, including involucrin and loricrin, are produced as skin matures imparting biomechanical strength to the epidermis [19, 20].

#### 2.7.2. Immunohistochemistry Examination

After tissue sections were dewaxed and rehydrated conventionally, sections were incubated with 3% H_2_O_2_ for 30 minutes. The slides were washed with PBS (pH 7.4) twice. The sections were blocked with 5% BSA in TBS for 20 minutes. After the redundant liquid had been discarded, the sections were incubated with loricrin antibody (Abcam, Cambridge, UK) at 4°C overnight. After slides had been washed with PBS, the slides were incubated with rabbit secondary antibody for 1 hour, followed by incubation with streptavidin-HRP for 20 minutes. The antibody binding sites were visualized by incubation with DAB-H_2_O_2_ solution.

#### 2.7.3. Masson's Trichrome Staining

Sections were dewaxed and rehydrated conventionally, placed in Weigert's hematoxylin stain for 1 h, rinsed under lukewarm water for 5 min, immersed in Masson solution for 15 min, and rinsed in deionized water before placing in phosphomolybdic acid for 10 min. Subsequently, sections were immersed in 2% aniline blue for 15 min, rinsed in 1% acetic acid, 95% ethanol, and absolute ethanol in turn, immersed in xylene for 10 min, and mounted with resin. Collagen fibers were stained blue, cytoplasm and erythrocyte were stained red, and nuclei were stained bluish brown.

#### 2.7.4. TUNEL Assay

Apoptosis assay was performed using the TACS.XL DAB In Situ Apoptosis Detection Kit (Trevigen, Gaithersburg, MD, USA). Briefly, sections were blocked by incubation in 3% H_2_O_2_ in methanol for 5 minutes at 25°C. Then the sections were labeled with TdT labeling reaction mix at 37°C for 1 h. Nuclei exhibiting DNA fragmentation were visualized by incubation in 3′,3-diaminobenzidine (DAB) for 15 min.

### 2.8. Statistical Analysis

All values are expressed as mean ± SEM. For comparisons of parametric data, one-way analysis of variance and then the Student's unpaired *t*-test were conducted. *P <* 0.05 was recognized to indicate statistical significance. For nonparametric data, Kruskal-Wallis test with Dunn's posttest was done. 

## 3. Results

### 3.1. Antioxidant Abilities in Antioxidant Sol-Gel

**Figure 1:**
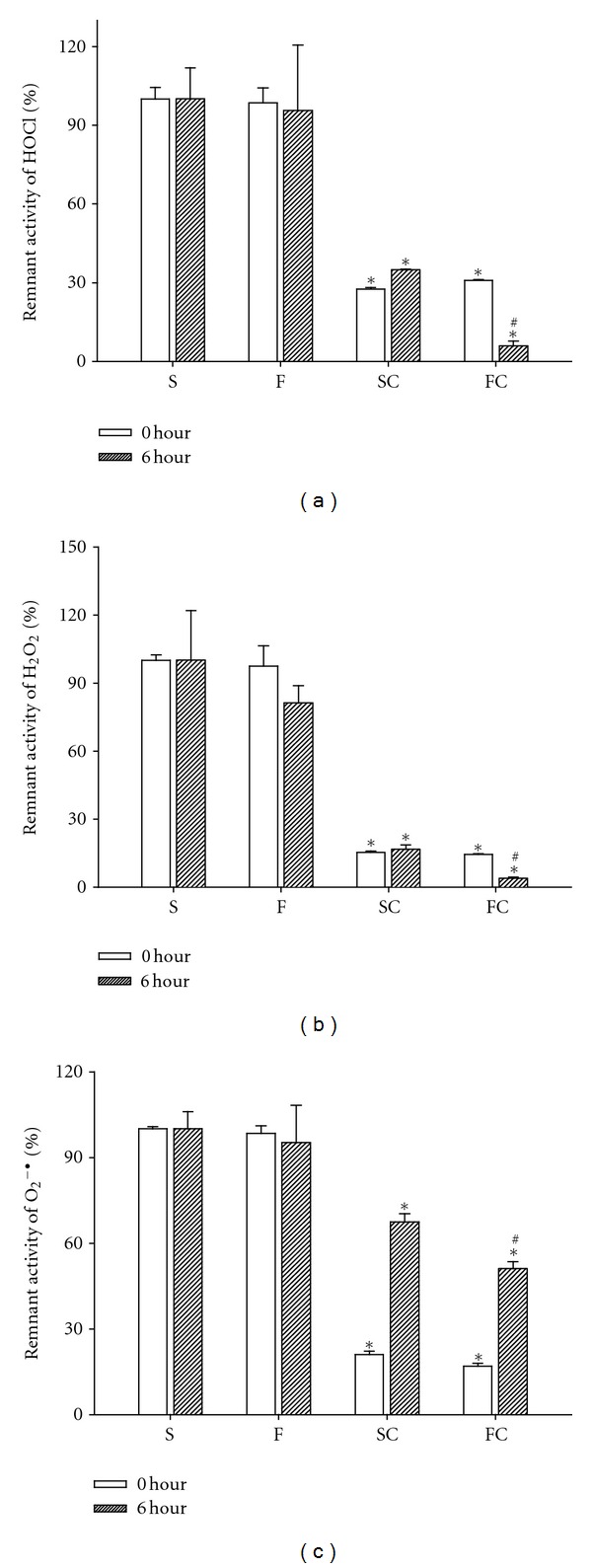
Scavenging abilities of saline (S), Pluronic F127 (F), antioxidant saline (SC), and antioxidant sol-gel (FC) for HOCl (a), H_2_O_2_ (b), and O_2_^−^ (c) after 0 and 6 hours of preparation. Fresh-prepared (0 hour) antioxidant saline and antioxidant sol-gel significantly decreased HOCl, H_2_O_2_, and O_2_^−^ counts when compared to saline. After 6 hours of air exposure, the antioxidant ability is significantly reserved in FC group when compared to SC group. Data are expressed as mean ± SEM. *P < 0.05, ^#^ *P <* 0.01 when compared to saline group. S: saline control; F: Pluronic F127; SC: saline plus vitamin C; FC: Pluronic F127 plus vitamin C.

We showed that the CL counts of H_2_O_2_, HOCl, and O_2_^−^ in Pluronic F127 and saline were similar, whereas the ROS levels in antioxidant sol-gel and antioxidant saline significantly decreased at 0 or 6 hours of air exposure (Figure 1). Our data indicated that the antioxidant sol-gel and antioxidant saline, not Pluronic F127, can directly scavenge ROS including H_2_O_2_, HOCl, and O_2_^−^. In addition, after 6 hours of air exposure, the antioxidant activities (except for O_2_^−^) in antioxidant sol-gel are stronger than those in antioxidant saline. This data implicates that Pluronic F127 can preserve parts of the antioxidant activities of vitamin C within after air exposure for a time period of such as 6 hours.

### 3.2. Wound Closure

**Figure 2:**
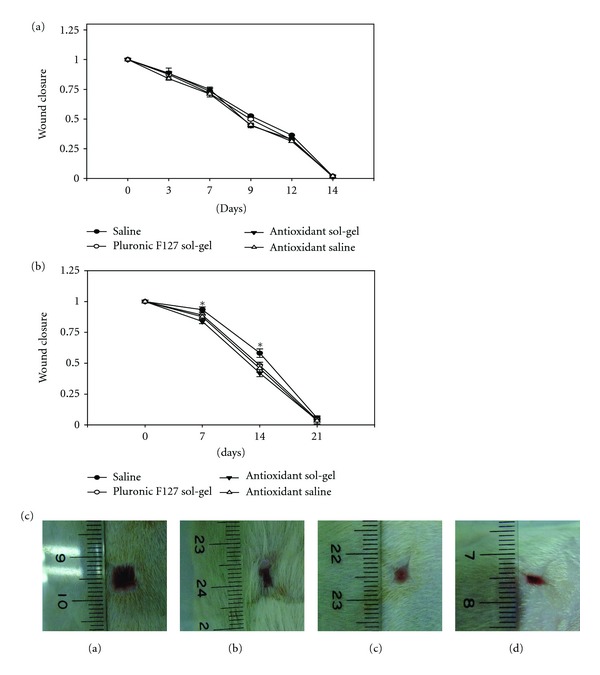
The effect of saline, Pluronic F127, antioxidant saline, and antioxidant Pluronic F127 on wound closure in the normal rats (a) and diabetic rats (b). Full-thickness skin wounds of 1.0 × 1.0 cm were measured from the time of wounding until closures. The skin defect was compared to the initial wound size to determine wound closure rate by tracing the wound. (a) Closure of full-thickness skin wounds of normal rats showed no significant difference in the healing rate between four groups of treatment. (b) Wound closure of diabetic skin showed that the antioxidant sol-gel-treated wounds closed faster than the saline-, sol- and antioxidant saline-treated wounds on days 7 and 14. Data is expressed as mean ± SEM for three separate experiments, each in quadruplicate. *P < 0.05 when compared to saline control. (c) Representative pictures of skin wounds in group saline (a), Pluronic F127 (b), antioxidant saline (c), and antioxidant sol-gel (d) at day 7.

Rats receiving STZ have significant elevation of blood glucose level (>300 mg/dL) after 3 weeks, which was sustained throughout the duration of the study. The wound healing of various treatments was evaluated in a full-thickness wound model. The wounds decreased in size gradually with time, closed at 2 weeks in normal rats and at 3 weeks in diabetic rats. We did not note any statistical difference in the wound closure of the normal rats with four kinds of treatment (Figure 2(a)). This was not unexpected since skin wounds of rats are known to heal efficiently and there is little room for improvement. However, in the diabetic rats, periodical observation of animals at 7 days showed a significant increase (*P <* 0.05) in the rate of contraction of wounds in the antioxidant sol-gel and antioxidant saline groups when compared to saline group (Figure 2(b)). In the diabetic rats at 14 days, a significant (*P <* 0.05) wound closure was noted in the pluronic F127, antioxidant sol-gel, and antioxidant saline groups when compared to saline group (Figure 2(b)). However, the wound closure rate was displayed in a tendency of antioxidant sol-gel > antioxidant saline = Pluronic F127 ≥ saline in the diabetic wounds at 14 days after wounding. However, all groups attained full closure by the end of the third week.

### 3.3. ROS Amounts in the Diabetic Skins

**Figure 3:**
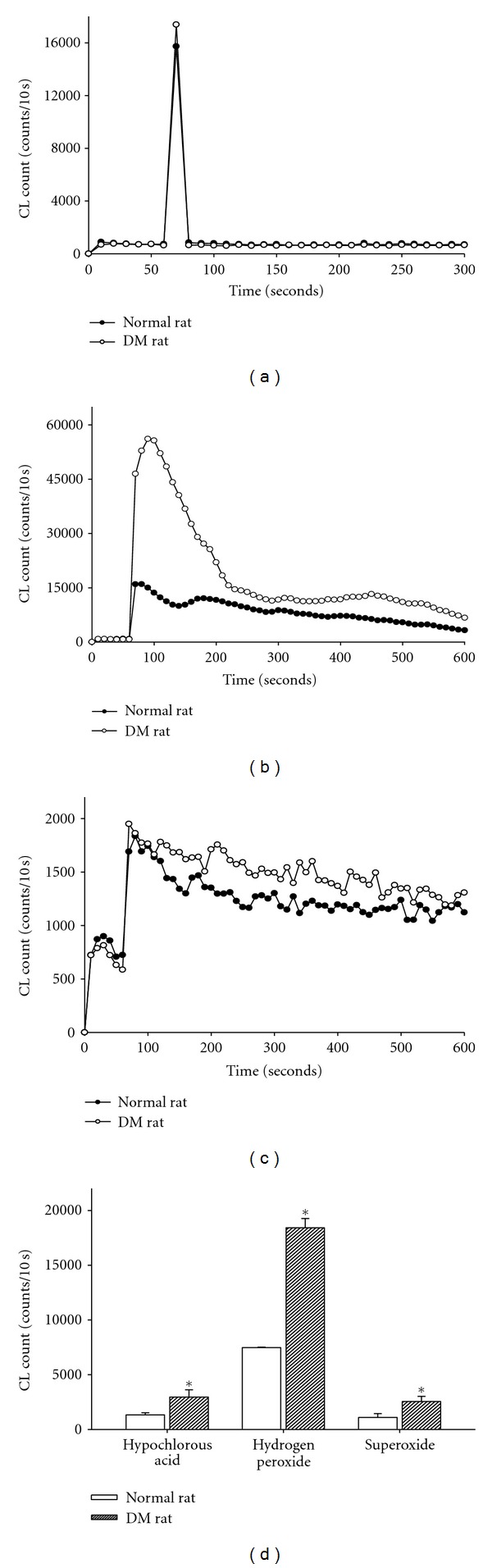
Representative data of HOCl, H_2_O_2_, and O_2_^−^ contents are measured in the normal and diabetic rat skins. The level of HOCl (a), H_2_O_2_ (b), and O_2_^−^ (c) counts is higher in the skin of diabetic rats than the normal rats. A summary data (d) shows that increased HOCl, H_2_O_2_, and O_2_^−^ counts are found in diabetic rats when compared to the normal rats. Data are expressed as mean ± SEM in 5 rats each. *P < 0.05 when compared to normal rats.

As shown in Figure 3, three types of ROS including H_2_O_2_, HOCl, and O_2_^−^ were all significantly increased in the intact skin of diabetic rats when compared with those in the skin of normal rats. These results directly evidence that diabetes increased oxidative stress in the skin before wounding.

### 3.4. Effect of Antioxidant Sol-Gel on Epidermal Maturation

**Figure 4:**
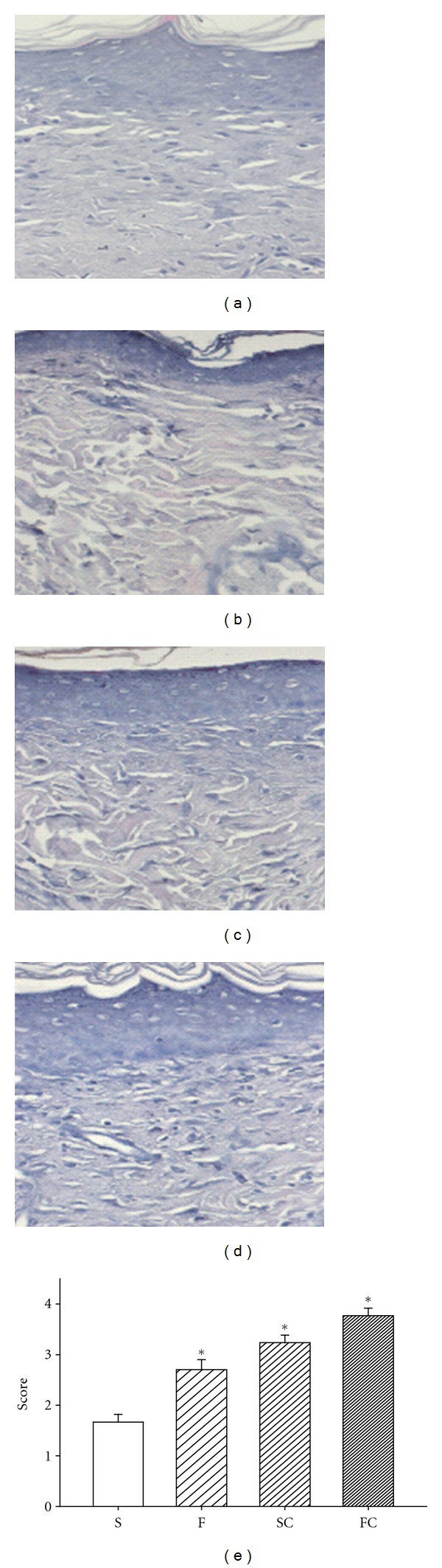
H & E stain in the saline control (a), Pluronic F127 (b), saline plus vitamin C (c), and Pluronic F127 plus vitamin C (d). Epidermal maturation was scored histologically from “no migration” (0) to “completed migration with keratinization” (4). The mean score of epidermal maturation is displayed in (e). Significant epidermal maturation indicated by migration of keratinization was shown here in the groups of SC and FC when compared with the group of Saline 14 days after wounding (e). Data are expressed as mean ± SEM. *Four groups are significantly different when compared with Kruskal-Wallis test and posttest comparing all pairs of columns. S: saline control; F: Pluronic F127; SC: saline plus vitamin C; FC: Pluronic F127 plus vitamin C. Original magnifications taken at ×200.

With the help of hematoxylin & eosin stain (Figure 4) and the epidermal differentiation marker loricrin immunohistochemistry (Figure 7), the accelerated healing was noted in the epidermis of antioxidant saline or sol-gel-treated groups. Significant epidermal maturation indicated by migration of keratinization is indicated in an order of antioxidant sol-gel > antioxidant saline > Pluronic F127 > saline. Loricrin was abundant and finely granular in cells of the granular layer. The labeling was maximally present in the upper granular cells and suddenly decreased in the cornified layer, where a brick wall-like staining resulted.

### 3.5. Effect of Antioxidant Sol-Gel on Dermal Maturation

**Figure 5:**
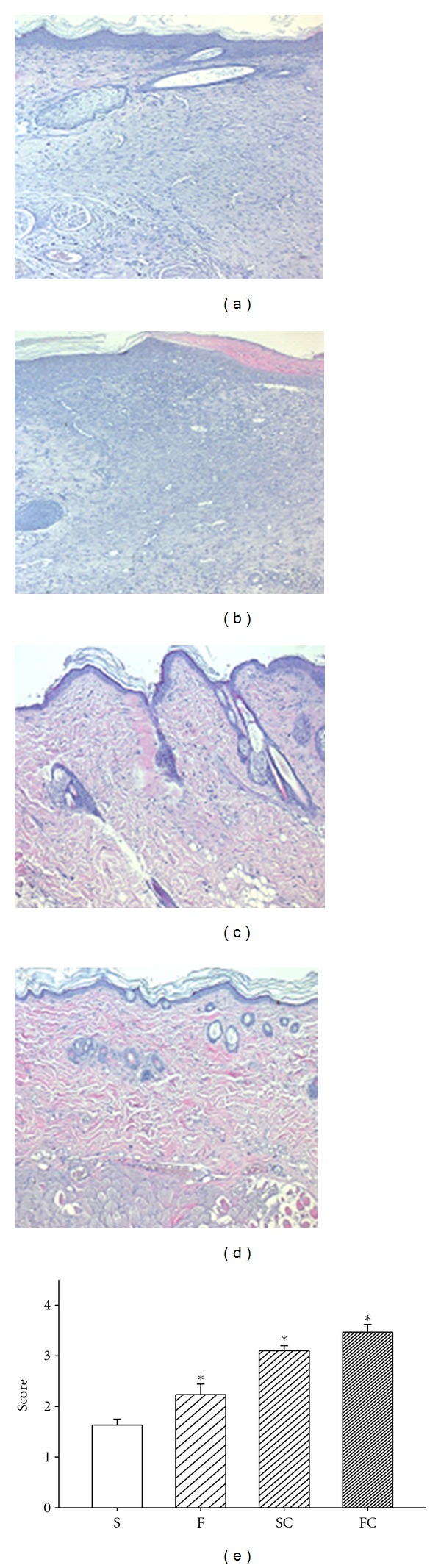
Effect of vitamin C on dermal maturation in diabetic rat wounds. Sections stained with H & E are displayed in the saline control (a), Pluronic F127 sol (b), saline plus vitamin C (c), and Pluronic F127 plus vitamin C (d). Histological evaluation of the 14-day wounds by H & E stain demonstrated enhanced healing characteristics including wound of proliferation, remodeling, and maturation in the antioxidant saline (c) or antioxidant sol-treated wound (d). Saline (a) or Pluronic 127 treatment (b) did not show any marked healing responses in the diabetic wounds. This advancement correlates with the fibroblast infiltration into the wounded area which was scored based on their maturity from reactive to normal. The mean score of dermal maturation is displayed in (e). The degree of dermal maturation is demonstrated in an order of FC > SC > F > S 14 days after wounding (e). Data are expressed as mean ± SEM. *Four groups are significantly different but not significant between saline and F127; vitamin C in saline and vitamin C in PF127. S: saline control; F: Pluronic F127; SC: saline plus vitamin C; FC: Pluronic F127 plus vitamin C. Original magnifications taken at ×100.

Normally, dermal recovery is assessed for three stages: proliferation, remodeling, and maturation. Histopathological examination with hematoxylin & eosin staining showed that the antioxidants sol-gel-treated wounds exhibited advancement in all these three stages. The histologic expression showed that dermal maturation was ranked in an order of antioxidant sol-gel > antioxidant saline > Pluronic F127 > saline (Figure 5(e)).

### 3.6. Effect of Antioxidant Sol-Gel on Masson's Trichrome Stain

**Figure 6:**
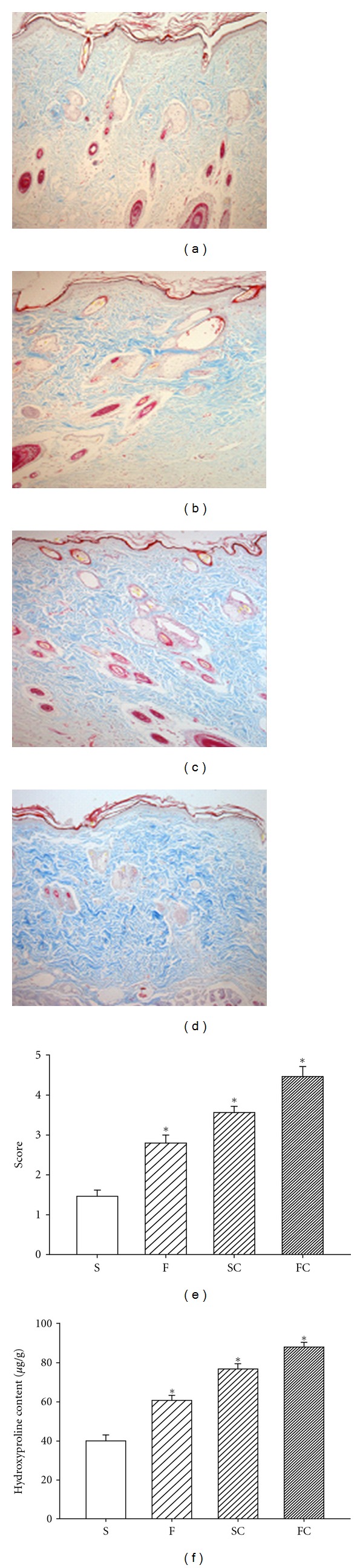
Effect of antioxidant sol-gel on collagen expression and collagen content at 14th day. Masson's trichrome staining of collagen in the saline control (a), Pluronic F127 sol (b), saline plus vitamin C (c), and Pluronic F127 plus vitamin C (d). The mean score of blue stain is displayed in (e). Four groups are significantly different when compared with Kruskal-Wallis test and posttest comparing all pairs of columns. Significant blue stain was demonstrated in an order of FC > SC > F > S. Increased hydroxyproline content was consistently increased in an order of FC > SC > F > S 14 days after wounding (f). Data are expressed as mean ± SEM. *P < 0.05 when compared to saline group. S: saline control; F: Pluronic F127; SC: saline plus vitamin C; FC: Pluronic F127 plus vitamin C. Original magnifications taken at ×200.

**Figure 7:**
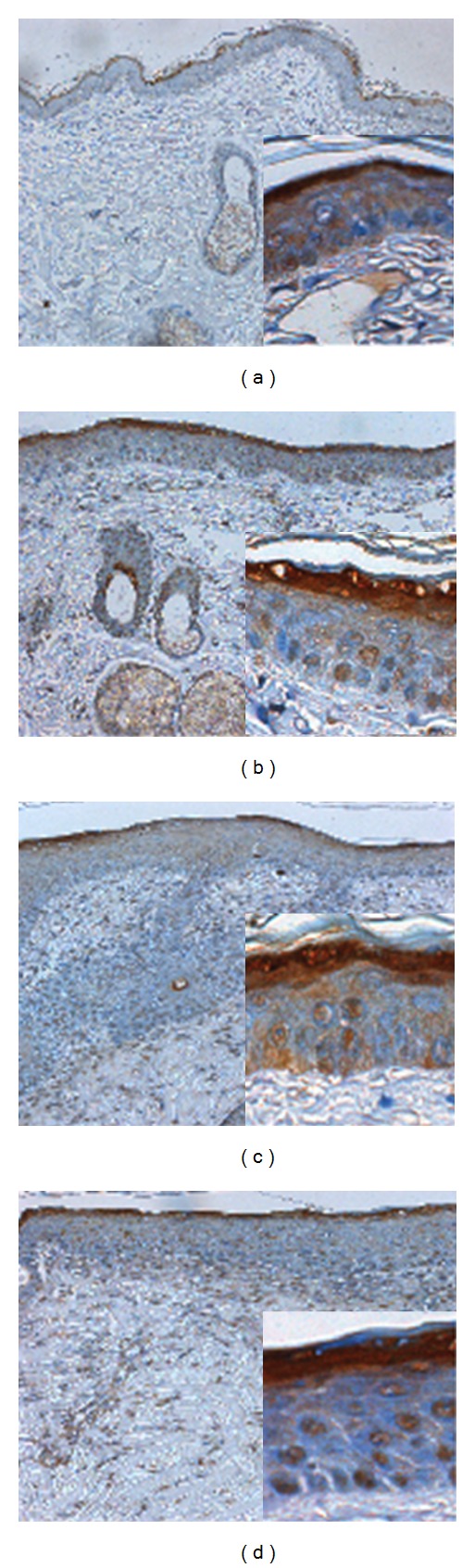
Immunohistochemical evidence of keratinization by loricrin at 14th day. Wounds treated with saline control (a), Pluronic F127 sol (b), saline plus vitamin C (c), and Pluronic F127 plus vitamin C (d) were demonstrated. Original magnifications taken at ×100 and ×200 as inset. Immunohistochemical staining by antiloricrin (epidermal differentiation marker) antibodies showed that loricrin was highly expressed in the upper granular cell layer, especially in the regenerated epidermis of Pluronic F127 plus vitamin C or saline plus vitamin C groups.

Collagen deposition and cellular proliferation can be measured in the histological cross-sections of wounds with Masson's trichrome staining. Significant increase in blue collagen stain was found in the antioxidant sol-gel-treated group (Figure 6(d)) and antioxidant saline-treated group (Figure 6(c)) when compared to saline-treated group (Figure 6(a)).

### 3.7. Hydroxyproline Estimation

Hydroxyproline is a major component of the protein collagen. Therefore, hydroxyproline content was used as an indicator to determine collagen content. As shown in Figure 6(f), hydroxyproline contents were significantly increased in the groups treated with antioxidant sol-gel (89.1 ± 2.3 *μ*g/mg), antioxidant saline (78.2 ± 12.6 *μ*g/mg), and Pluronic F127 sol group (60.5 ± 1.2 *μ*g/mg) when compared with saline group (40.1 ± 3.1 *μ*g/mg).

### 3.8. TUNEL Study

**Figure 8:**
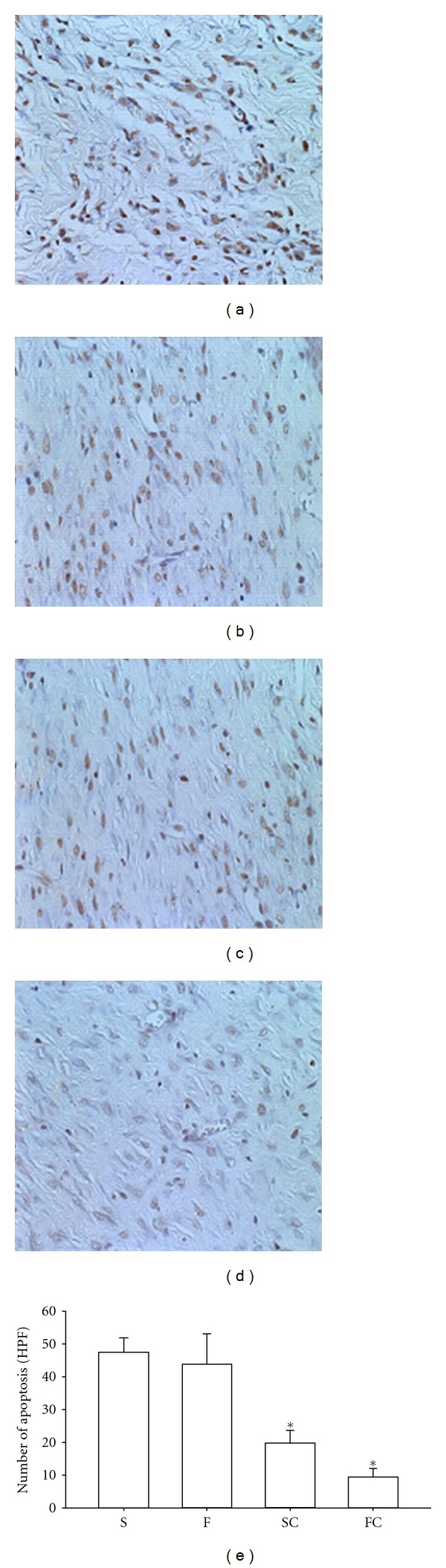
Effect of antioxidant sol-gel on apoptosis production of the healing skin in diabetic rats at 7th day. Apoptosis analyzed by TUNEL stain (brown color in the nucleus) was shown in the saline control (a), Pluronic F127 sol (b), saline plus vitamin C (c), and Pluronic F127 plus vitamin C (d). The data were expressed as the number of apoptosis (high power field) in each section (400x) is displayed in (e). The percentage of apoptosis appearance in the wounds is demonstrated in an order of FC > SC > F > S 14 days after wounding (e). Data are expressed as mean ± SEM. *P < 0.05 when compared to saline group. S: saline control; F: Pluronic F127; SC: saline plus vitamin C; FC: Pluronic F127 plus vitamin C. Original magnifications taken at ×200.

The apoptosis formation analyzed by TUNEL stain showed that a marked increase of apoptosis in the wound tissue of diabetic rats treated with topical saline 7 days, after injury. The topical application of antioxidant sol-gel or antioxidant saline significantly decreased the apoptosis production in the diabetic wounds (Figure 8). The potential of inhibiting apoptosis production was expressed in the order of antioxidant sol-gel > antioxidant saline > Pluronic F127 > saline (Figure 8(e)).

## 4. Discussion

The present study showed that the effect of *in vitro* drug release profiles indirectly indicated by the antioxidant activities demonstrated that the vitamin C from the Pluronic F127 was continuously released to depress H_2_O_2_, HOCl, and O_2_^−^ amounts after 6 hours of air exposure at 37°C. The *in vivo* study further indicated that continuous release of vitamin C by using Pluronic F127 as a drug delivery vehicle exerted efficiently therapeutic potential on diabetic wound healing via its antioxidant and antiapoptotic effects. The antioxidant sol-gel is better than antioxidant saline in scavenging ROS, promoting collagen synthesis, epidermal and dermal maturation, and decreasing apoptosis production in the diabetic wound.

Pluronics (also called Poloxamers) have been particularly interesting because this polymer shows a critical solution temperature (reverse sol-gel transition temperature) below the human physiological temperature and, thus, exists to a gel state in the body at 37°C. Yamaoka et al. [21] indicated that the copolymer films are biocompatible materials with controllable mechanical properties and biodegradability. In addition, Pluronic F127 caused relatively low inflammatory response and showed nontoxicity, and thus could be a good candidate material as a coatable wound dressing gel [22]. Hokett et al. [23] and Fowler et al. [24] had used Pluronics to ameliorate the wound-healing process in gingival and bony wounds, respectively. In the report of Khalil et al. [25] and also our study, normal rats treated with the Pluronic F127 alone showed results similar to the saline control animals without improving the wound-healing process. A 10% (w/w) Pluronic F127 has been added to the Jordanian traditional medicinal plants to modify the aqueous extract viscosity and to stabilize the oil dispersion. The applied Pluronic F127 continuously released the Jordanian traditional medicinal plants aqueous extract and significantly promoted the wound-healing process [25]. In our study, Pluronic F127 vehicle alone coating on wound did not show any inflammatory symptoms or toxicity and did not affect wound-healing process in the normal rats. This finding is consistent with a previous study [25]. As a drug delivery vehicle, we found that continuous vitamin C release from Pluronic F127 vehicle can partly sustain the scavenging ability against H_2_O_2_, HOCl, and O_2_^−^ amount after 6 hours of air exposure.

Wound healing is a complex multifactorial process that results in the contraction and closure of the wound and restoration of a functional barrier. One of the leading causes of impaired wound healing is diabetes mellitus. In diabetic rats, a minor skin wound often leads to chronic, nonhealing ulcers and ultimately results in gangrene, even amputation. ROS and oxidative stress arise from inflammatory cells, which are strongly implicated in the pathogenesis of several diseases including chronic ulcers [26–28]. Rasik and Shukla [29] reported the decrease in antioxidants and the increase in oxidative stress delaying healing in excision cutaneous wounds in diabetic, aged, and immunocompromised animals. They further showed that skin levels of catalase, glutathione, vitamin C, and vitamin E in streptozotocin-induced diabetic rat were lower as compared to nondiabetics. In chronic wounds, fibroblast dysfunctions, such as increased apoptosis, premature senescence, senescence-like phenotype, or poor growth response in the absence of senescence markers may be due to excessive amounts of oxidative stress [4]. Our evidence in Figure 3 directly demonstrated that the skin levels of H_2_O_2_, HOCl, and O_2_^−^ are significantly increased in the diabetic skin adjacent to the wounds when compared to the control skin of normal rats.

Several studies from rat dermal wound have shown that the treatment of antioxidants to depress ROS is beneficial for wound healing. An improvement in the quality of wound healing has been attempted by slow delivery of antioxidants-like curcumin from collagen, which also acts as a supportive matrix for the regenerative tissue [30]. Biochemical parameters and histological analysis revealed that curcumin-incorporated collagen films increased wound reduction and enhanced cell proliferation and efficient free radical scavenging [30]. Another study [31] has shown that topical application of resveratrol accelerated wound contraction and closure associated with a more well-defined hyperproliferative epithelial region, higher cell density, enhanced deposition of connective tissue, and improved histological architecture. Study bySilvetti [10] has presented a safe and effective method of improving repair and controlling infection of wounds by daily topical application of a balanced solution of salts, amino acids, a high-molecular weight, D-glucose polysaccharide, and vitamin C. In skin, vitamin C has growth factor-like properties and is an important regulator for collagen synthesis of the extracellular matrix [8]. Vitamin C appears capable of overcoming the reduced proliferative capacity of elderly dermal fibroblasts, as well as increasing collagen synthesis in elderly cells by similar degrees as in newborn cells even though basal levels of collagen synthesis are age dependent [8]. By correcting a defect (underhydroxylation) in a posttranslational event and by increasing collagen production, dietary ascorbic acid improved the collagen status of a diabetes-perturbed connective tissue [5]. Our data from normal rats, with or without antioxidant showed no difference in wound closure rate. However, wound closure rates increased significantly in antioxidant sol-gel and antioxidant saline groups of the diabetic rats, indicating that increased vitamin C supplement improved the wound-healing process. We assumed that this therapeutic effect of vitamin C on diabetic wounds may be due to its inhibition of excess ROS production. This hypothesis was further supported by our finding that a significant increase of ROS including H_2_O_2_, HOCl, and O_2_^−^ was found in the diabetic skins.

ROS can affect proliferative and cell survival signaling to alter apoptotic pathways in the skin diseases. Excess production of ROS in the skin can foster the development of dermatological diseases. One approach to prevent or treat these ROS-mediated disorders is based on the administration of various antioxidants in an effort to restore homeostasis. Many antioxidants have shown substantive efficacy in cell culture systems and in animal models of oxidant injury. On the other hand, increased apoptosis formation delayed wound healing [32]. Diabetes caused more than twofold induction of 71 genes that directly or indirectly regulate apoptosis and significantly enhanced several caspases activity, and inhibiting apoptosis significantly improved several parameters of healing, including fibroblast density, enhanced mRNA levels of collagen I and III, and increased matrix formation [33]. This means that diabetes-enhanced apoptosis represents an important mechanism through which healing is impaired partly by diabetes-increased expression of proapoptotic genes and caspase activity. Fadini et al. [32] demonstrated that diabetes induced p66Shc expression and activation, subsequently produced H_2_O_2_ and delayed wound healing in mice with reduced granulation tissue thickness and vascularity, and increased apoptosis. They further found that the use of p66Shc knockout mice associated with a less enhancement of oxidative stress improved wound healing in diabetic animals. Therefore, a reduction of oxidative stress may reduce apoptosis formation and improve the impairment of wound-healing process in diabetics. Based on our data, the antioxidant sol showed a strong scavenging activity for ROS and depressed diabetes-evoked apoptosis formation. This antioxidant sol clearly hastened the wound-healing process with the increased collagen synthesis, enhanced epidermal and dermal maturation, and the decreased apoptosis formation.

## 5. Conclusions

We demonstrate that vitamin C incorporated into Pluronic F127 exerts continuous effects of antioxidant and anti-apoptotic activities, which enhance epidermal and dermal maturation and collagen synthesis in the diabetic skins. The antioxidant sol-gel can decrease potentially harmful factors such as ROS production and apoptosis cell death present in chronic wound of the diabetic rats. These characteristics suggest a beneficial role for this preparation in helping rebalance the chronic wound environment and therefore promote healing.
